# Plasma fibrinogen level and acute aortic dissection prognosis—insights from a two-center cohort study

**DOI:** 10.3389/fcvm.2025.1508749

**Published:** 2025-09-23

**Authors:** Jiaxin Xiao, Junshuang Tang, Zilong Fu, Kaihong Yi, Xiulian Deng, Junsi Zheng, Qingqing Ni, Shiwan Wu, Yandan Xie, Weixing Huang, Yongquan Zhang, Xiao Wang, Liang Tao, Yequn Chen, Muli Wu

**Affiliations:** ^1^ Department of Cardiology, First Affiliated Hospital of Shantou University Medical College, Shantou, Guangdong, China; ^2^ Shantou University Medical College, Shantou, Guangdong, China; ^3^ Second Affiliated Hospital of Xi'an Medical College, Xi'an, Shanxi, China; ^4^ Department of Cardiac Surgery, Wuhan Asia Heart Hospital Affiliated with Wuhan University of Science and Technology, Wuhan, Hubei, China

**Keywords:** fibrinogen, acute aortic dissection, two-center study, meta-analysis, prognosis

## Abstract

**Objective:**

The relationship between plasma fibrinogen level (PFL) and prognosis of acute aortic dissection (AAD) are not well defined. The present study aimed to assess the effect of PFL on AAD prognosis through a two-center study and meta-analysis.

**Methods:**

A two-center cohort study was carried out in the two hospitals from Shantou and Xi'an cities. 1981 patients with AAD, admitted from 2012 to 2021, were included and followed up by clinical interview and telephone. The primary follow-up outcomes were 30-day mortality and long-term mortality. The relationship between PFL and all-cause mortality was identified. Further, meta-analysis was performed using our data and open access data.

**Results:**

The median follow-up time for the study cohort was 21.6 months (interquartile range 8.6–45.4 months). Compared with survivors, the non-survivors had a lower PFL. Survival analysis showed that mortality was higher in those with lower PFL. After multivariate adjustment, each 1 g/L increase in PFL was associated with a 18.9% decrease in 30-day mortality rate and a 11.5% decrease in long-term mortality rate (*P* < 0.001). Meta-analysis of the included our study revealed a significant association between lower PFL and increased 30-day mortality in type A and type B AAD [OR = 3.30, 95% CI: 2.58–4.23, *P* = 0.0739; *I*^2^ = 47.9%]. Simultaneously, similar associations were observed in Stanford type A in for long-term mortality [OR = 3.62, 95% CI: 2.23–5.87, *P* = 0.0438; *I*^2^ = 56.2%].

**Conclusions:**

Low PFL is a risk factor for short and long-term all-cause mortality in patients with type A AAD and short-term all-cause mortality in patients with type B AAD.

## 1 Introduction

Acute aortic dissection (AAD) is an emergency with high mortality (1). The cause of aortic dissection is not yet clear, and predicting mortality risk and carrying out precision therapy is still challenging. Unfavorable prognostic factors for mortality of AAD include Stanford type A AAD, older age, abrupt onset of chest pain, hypotension/shock/cardiac tamponade and pulse deficit (2–4). Blood biochemical factors participate in the occurrence and development of aortic dissection and confer important value for prognostic prediction. The extrinsic pathway of the coagulation cascade is initiated by tissue factor exposure due to intimal tear in the aortic media, which subsequently triggers activation of fibrinolytic systems in AAD patients and participates in the repair of the dissection (1). Previous studies have identified an increase in plasma D-dimer concentration, a degradation product of cross-linked fibrin, as a potential serum marker for AAD (2–5). In recent years, a limited number of studies have explored the relationship between PFL and risk of in-hospital mortality among Stanford type A AAD patients, and the results are inconsistent. It is still unclear whether PFL, an important component of the coagulation system, is involved in the occurrence and development of aortic dissection, as well as impacts the prognosis of AAD (6, 7). Therefore, we conducted a two-center cohort and meta-analysis study to evaluate the association of PFL and all-cause mortality of AAD.

## 2 Methods

### 2.1 Two-center cohort study

**Figure 1:**
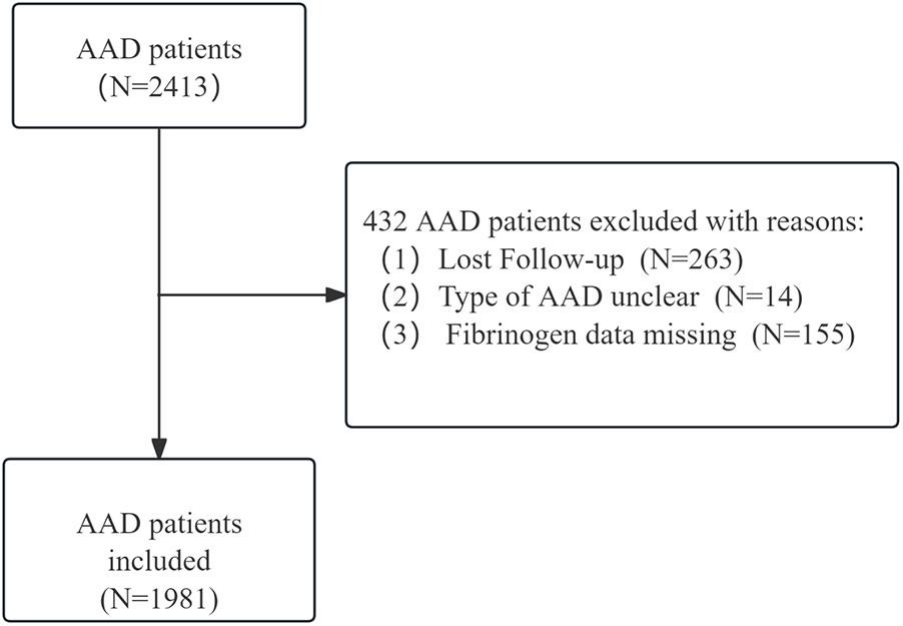
Flow-chart for study selection and exclusion of patients with AAD.

Consecutive patients diagnosed with AAD were recruited from January in 2012 to December in 2021 for the cohort (8, 9) of acute aortic dissection patients in the First Affiliated Hospital of Shantou University Medical College in Shantou, Second Affiliated Hospital of Xi'an Medical College. AAD was defined as a separation of the layers of the aortic wall due to an intimal tear within two weeks from initial clinical presentation. Computed tomographic angiography was employed to definitively diagnose aortic dissection, and its categorization was determined using the Stanford classification system. Specifically, Stanford type A aortic dissection is that includes the ascending aorta, regardless of intimal tear's site and distal extension of the aortic lesion. And Stanford type B is referred to involved the descending thoracic aorta distal to the left subclavian artery and its distal end. Within the two-center aortic dissection cohort study, a total of 2,413 patients were identified as having AAD. 14 patients had an unclear type of aortic dissection, 263 patients were lost to follow-up, and a further 155 patients had incomplete serum fibrinogen data, as illustrated in Figure 1. Ultimately, the study included 1981 patients diagnosed with AAD. Ethical approval for the study was obtained from the ethics committee of the First Affiliated Hospital of Shantou University Medical College. This cohort study adhered to the Declaration of Helsinki, and was constructed based on the STROBE cohort reporting guidelines.

### 2.2 Data collection

In-hospital data was collected from medical records. In order to reduce selected bias risk, we exclusively collected PFL on the first day of the hospital admission and excluded patients diagnosed with aortic penetrating ulcer or chronic aortic dissection. Age, gender, type of AAD, surgery for type A AAD or endovascular stent-graft implantation for type B AAD, and history of hypertension, diabetes mellitus and coronary heart disease(CHD), international normalized ratio of prothrombin time (PT-INR) and plasma D-dimer concentration were collected. After discharge, follow-up was performed, by clinical interview or telephone, at 1, 3, 6, and 12 months, and every year after that. The primary outcome was 30-day all-cause mortality and long-term all-cause mortality.

### 2.3 Meta-analysis

Literature searches were performed in PubMed Library up to May 2024 by using various combinations of keywords including “Aortic Dissection”, “Aortic Dissections”, “Dissection, Aortic”, “Aortic Dissecting Aneurysm”, “Aneurysm, Aortic Dissecting”, “Aortic Dissecting Aneurysms”, “Dissecting Aneurysm, Aortic”, “Dissecting Aneurysm Aorta”, “Aneurysm Aorta, Dissecting”, “Aneurysm Aorta, Dissecting”, “Aorta, Dissecting Aneurysm”, “Dissecting Aneurysm Aortas”, “Aneurysm, Dissecting”, “Dissecting Aneurysms”, “Dissecting Aneurysm”, “Fibrinogen”, “Blood Coagulation Factor I”, “Coagulation Factor I”, “Factor I, Coagulation”, “Factor I”, “gamma-Fibrinogen” and “gamma Fibrinogen”. In addition, we manually searched the references of the retrieved literature in order to avoid missing potential eligible studies. The outcome for this analysis was AAD mortality. The articles were included if they: (1) were described in English, (2) compared the association of PFL and mortality in a cohort study, and (3) reported relative risks with 95% CIs, or the data to calculate them. Studies where only the abstract was available were excluded. Finally, name of first author, year of publication, age, the content to which the subject belonged, sample size, OR (adjusted OR if provided) and 95%CI were extracted.

### 2.4 Statistical analysis

Patients were classified as survivors or non-survivors, with those lost to follow-up excluded from the analysis. Continuous variables were presented as mean/SD or median/interquartile range depending on the results of the Kolmogorov–Smirnov normality test, while categorical data were presented as numbers and proportions. Mann–Whitney *U* tests and chi-square tests were used to compare baseline characteristics between the two groups for continuous and categorical variables, respectively. The Cox proportional hazards model was used to calculate the hazard ratio and 95% CI for the association between PFL and AAD mortality risk. Three multivariate Cox models, adjusted for potential confounders, were established to determine whether PFL was an independent predictor of mortality. X-tile analysis (version 3.6.1) and Area Under the Receiver Operating Characteristic Curve(AUC) were used to identify the optimal PFL cut-off value for predicting AAD mortality risk (10). Kaplan–Meier survival curves, stratified by PFL cut-off and quartile-based categorization, were constructed using the log-rank test. Subgroup analyses of interaction were performed for age (>60 vs. ≤60), gender (female vs. male), hypertension (yes vs. no), diabetes (yes vs. no), and aortic dissection type (A vs. B). Statistical significance was considered at a threshold of *p* < 0.05. In the meta-analysis, the Newcastle-Ottawa Scale (nine-star system) was used to assess the quality of the included studies (11). The *I*^2^ statistic was used to quantify heterogeneity, with a fixed-effect model applied if *I*^2^ ≤ 50% and a random-effect model used otherwise. In addition, a sensitivity analysis was performed using a one-study-removed analysis. All analyses were conducted using R (version 4.3.3).

## 3 Results

### 3.1 Baseline clinical characteristics

This study included 1981 AAD patients, aged 62 on average, with 440 females (22.2%). The median follow-up was 21.6 months (interquartile range 8.6–45.4 months). For the two endpoint events, 294 patients died within 30 days, indicating a 14.8% 30-day mortality rate, and 487 patients died during the long-term follow-up, showing a 24.6% long-term mortality rate. The non-survivor group had a significantly higher average age and a larger proportion of Stanford type A dissection cases compared to the survivor group. PFL in the non-survivor group was also markedly lower (3.28, 2.16–4.28 g/L vs. 3.72, 2.59–4.73 g/L in the survivor group, *p* < 0.001) (Table 1).

**Table 1:** Baseline characteristics of the participants.

Characteristic	Overall	Survival	Non-survivor	*P*-value
	*N* = 1,981	*N* = 1,494	*N* = 487	
Age(years)	62 (53–70)	61 (52–69)	65 (56–73)	<0.001
Female gender *n* (%)	440 (22.2)	339 (22.7)	101 (20.7)	0.403
TBAD, *n* (%)	1,302 (65.7)	1,060 (71.0)	242 (49.7)	<0.001
Hypertension, *n* (%)	1,587 (80.1)	1,189 (79.6)	398 (81.7)	0.336
Diabetes, *n* (%)	549 (27.7)	449 (30.1)	100 (20.5)	<0.001
CHD, *n* (%)	263 (13.3)	201 (13.5)	62 (12.7)	0.74
Surgery, *n* (%)	393 (19.8)	328 (22.0)	65 (13.3)	<0.001
Cover stents, *n* (%)	729 (36.8)	625 (41.8)	104 (21.4)	<0.001
Fibrinogen (g/L)	3.56 (2.48–4.63)	3.72 (2.59–4.73)	3.28 (2.16–4.28)	<0.001
PT-INR	0.99 (0.93–1.06)	0.98 (0.92–1.05)	1.03 (0.95–1.13)	<0.001
DD (*μ*g/L)	2,350 (1,306–4,640)	2,100 (1,211–4,098)	3,700 (1,790–6,199)	<0.001

Kolmogorov–Smirnov tests of normality were performed for all quantitative values. The quantitative values (age, fibrinogen, PT-INR, DD) were not normally distributed, thus data are presented as the median (interquartile range). The *p*-value of continuous or categorical variables was obtained by the Mann–Whitney *U* test and chi-square test, respectively.

TAAD, Stanford type A AAD.

### 3.2 Association of PFL with mortality

Fibrinogen levels on admission are associated with all-cause mortality in patients with AAD.

**Figure 2:**
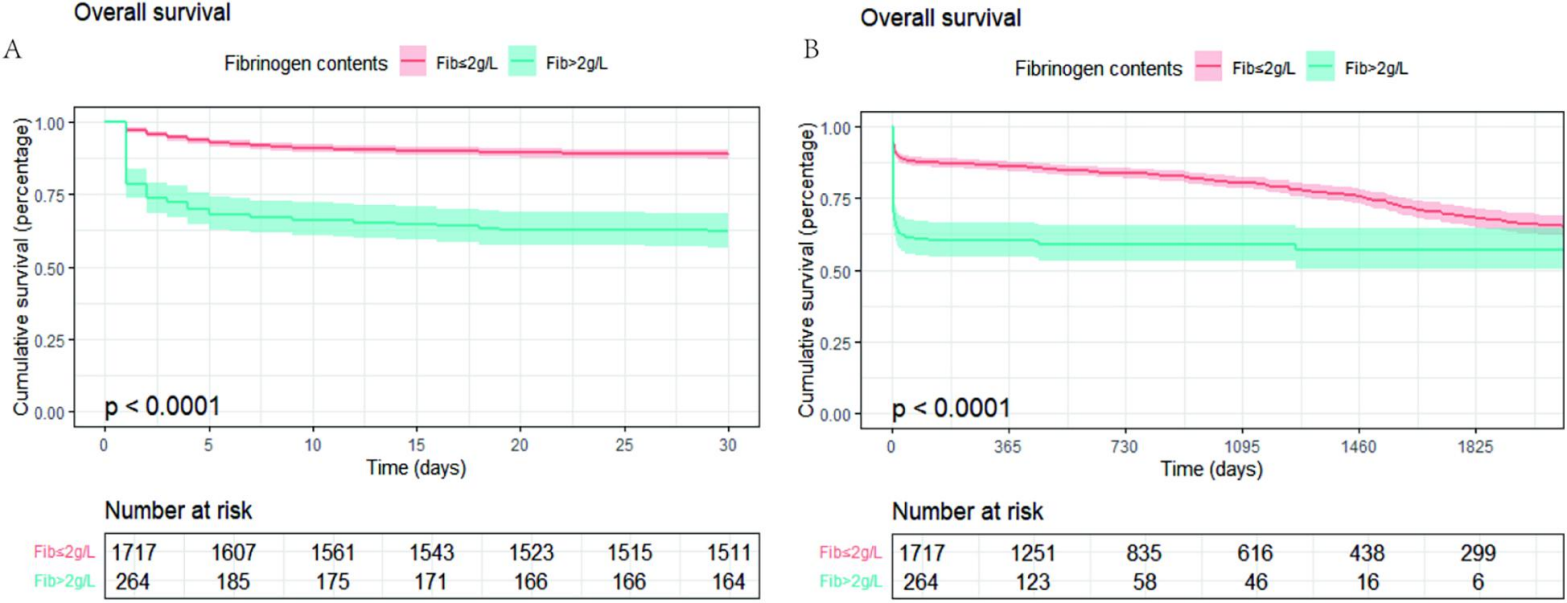
Kaplan–meier survival curves Kaplan–Meier survival curves for groups stratified by PFL ≤2.0 g/L or PFL >2.0 g/L. **(A)** 30-day mortality KM curve; **(B)** long-term mortality KM curve.

The optimal cutoff points determined by X-tile analysis and AUC curve were both 2.0 g/L (Supplementary Figures S1, S2). Patients were divided into higher (>2.0 g/L) and lower (≤2.0 g/L) PFL groups. Kaplan–Meier survival curve analysis showed that fibrinogen levels ≤2.0 g/L were associated with higher risk of all-cause mortality in AAD patients (*p* < 0.001, Figure 2). Using multivariate Cox proportional hazard analysis, three distinct models were constructed. In the unadjusted Cox proportional hazards model 1, low fibrinogen was a risk factor for long-term mortality (HR = 2.923, 95% CI: 2.353–3.632, *p* < 0.001). Model 2, adjusting for age and sex, low fibrinogen was a risk factor for long-term mortality (HR = 3.11, 95% CI: 2.499–3.870, *p* < 0.001). Moreover, in Model 3, which incorporated additional adjustments for region, hypertension, diabetes, CHD, type of AD, surgical operation, stent-grafting, and plasma D-dimer levels. For every 1 g/L increase in PFL, the 30—day mortality rate decreased by 18.9%, and the long—term mortality rate decreased by 11.5%.Compared with PFL > 2 g/L, the risk of 30-day all-cause mortality for PFL ≤ 2 g/L was (HR = 2.429, 95% CI: 1.879–3.141, *p* < 0.001), and the effect on long-term mortality was HR = 2.017, 95% CI: 1.591–2.556, *p* < 0.001 (Table 2).

**Table 2:** Associations of risk of mortality in AAD patients.

Variable	Model 1		Model 2		Model 3	
	HR (95%CI)	*P*-value	HR (95%CI)	*P*-value	HR (95%CI)	*P*-value
30-day mortality						
PFL per 1 g/L increase	0.603 (0.549–0.662)	<0.001	0.597 (0.543–0.656)	<0.001	0.811 (0.736–0.893)	<0.001
Dichotomous groups						
PFL > 2 g/L	Reference		Reference		Reference	
PFL ≤ 2 g/L	4.171 (3.276–5.311)	<0.001	4.287 (3.362–5.467)	<0.001	2.429 (1.879–3.141)	<0.001
Long-term mortality						
PFL per 1 g/L increase	0.717 (0.668–0.770)	<0.001	0.700 (0.652–0.753)	<0.001	0.885 (0.819–0.956)	0.002
Dichotomous groups						
PFL > 2 g/L	Reference		Reference		Reference	
PFL ≤ 2 g/L	2.923 (2.353–3.632)	<0.001	3.11 (2.499–3.870)	<0.001	2.017 (1.591–2.556)	<0.001

Model 1: unadjusted.

Model 2: adjusted for age, gender.

Model 3: adjusted for age, gender, hypertension, diabetes, CHD, surgery, types of AD, cover stents, DDI, region.

### 3.3 Subgroup analyses

**Figure 3:**
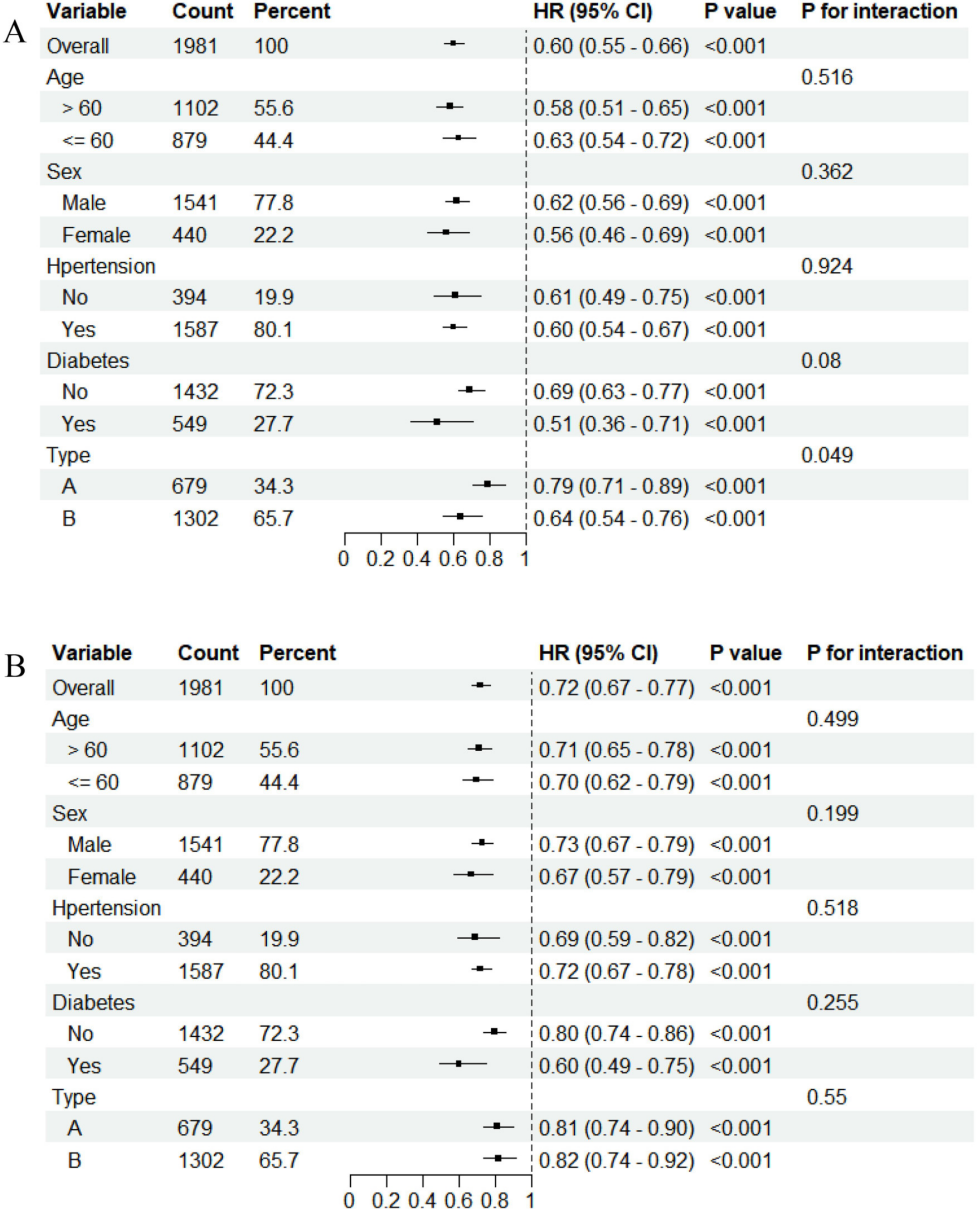
Subgroup analyses and interaction plots of the association between fibrinogen and mortality. **(A)** 30-day mortality. **(B)** long-mortality.

It showed the subgroup analysis of interaction between PFL and the risk of mortality in patients with aortic dissection (Figure 3). PFL was analyzed as a continuous variable, and the results indicated that low PFL was associated with an increased risk of all-cause mortality in patients with aortic dissection. No significant interaction was observed in the analysis of long-term mortality. However, a significant interaction between dissection type and PFL was noted in the analysis of 30-day mortality (*P* for interaction = 0.049). The hazard ratio (HR) for 30-day all-cause mortality was more pronounced in patients with type B dissection compared with those with type A dissection [HR: 0.64 (95% CI: 0.54–0.76) vs. HR: 0.79 (95% CI: 0.71–0.89)].

### 3.4 Meta-analysis

**Figure 4:**
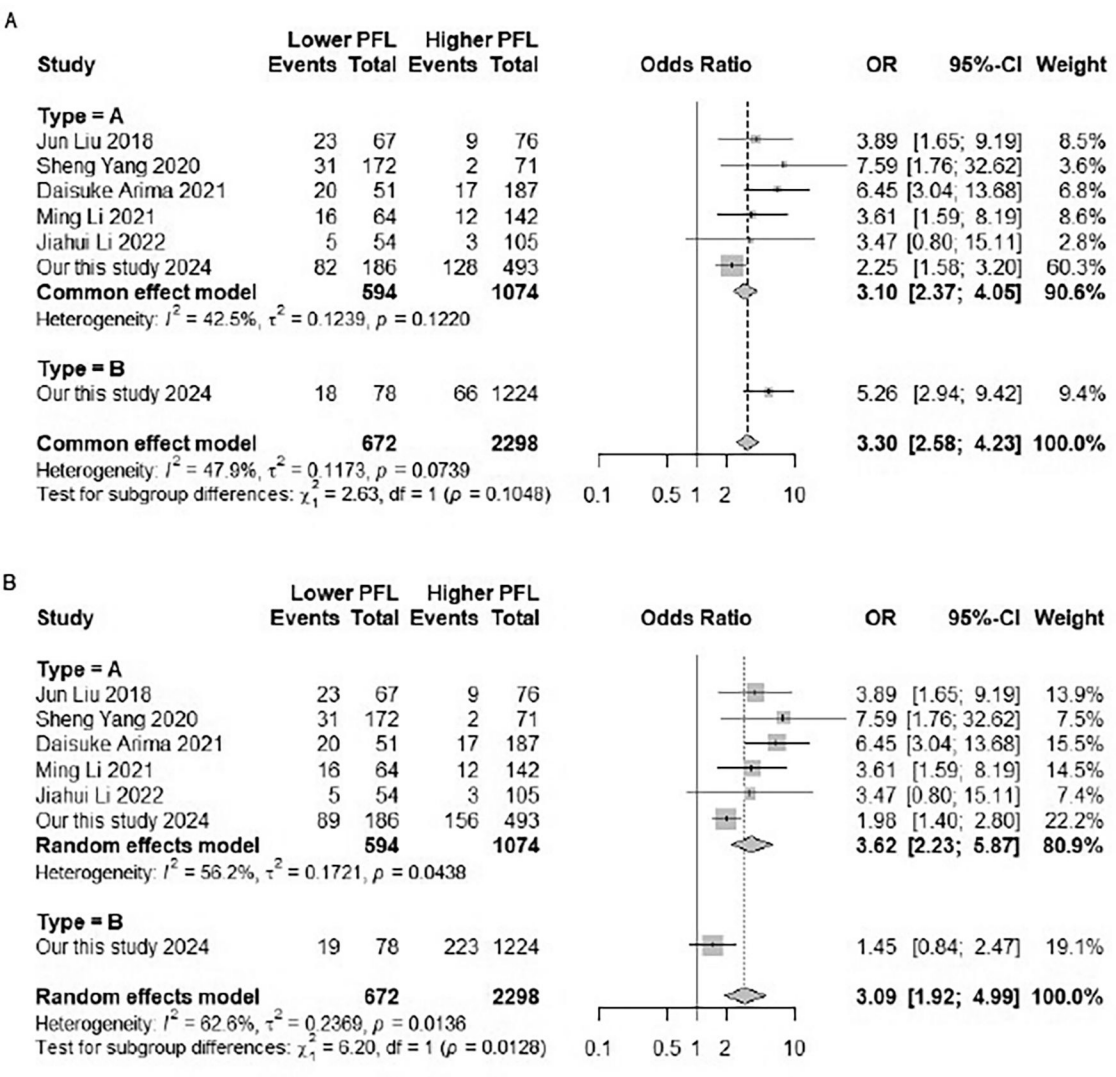
Forest plot of the relationship between PFL and mortality in patients with AAD. **(A)** Forest plot of subgroup analysis for in-hospital and 30-day mortality. **(B)** Forest plot of subgroup analysis for aortic dissection types A and B.

Supplementary Figure S2 showed the search strategy. According to the inclusion criteria, five studies involving 989 patients were eventually enrolled, with a total of 2,970 participants ultimately being included in the meta-analysis after including the participants of the present study (6, 7, 12–14). Table 3 describes the main features of the selected studies. Most of the studies were given more than ﬁve stars according to the Newcastle control study (Table 3) (15). To reduce heterogeneity among studies, we used 30-day mortality as the endpoint for the meta-analysis. The datasets were heterogeneous (*I*^2^ = 47.9%). Therefore, fixed effects models were used for analyses. Meta-analysis of the included studies and our study revealed a significant association between lower PFL and increased all-cause mortality [OR = 3.30, 95% CI: 2.58–4.23, *P* = 0.0739; *I*^2^ = 47.9%]. Meta-analysis of the included studies and our study revealed a significant association between lower PFL and increased 30-day mortality in type A and type B AAD [OR = 3.30, 95% CI: 2.58–4.23, *P* = 0.0739; *I*^2^ = 47.9%]. Simultaneously, similar associations were observed in Stanford type A in for long-term mortality [OR = 3.62, 95% CI: 2.23–5.87, *P* = 0.0438; *I*^2^ = 56.2%]. We could observe negative correlation trend in Stanford type B AAD patients, but it was not significant difference [OR = 1.45, 95% CI: 0.84–2.4] (Figure 4).

**Table 3:** Characteristics of included studies for meta-analysis.

Author, Year, region	Design	Cut off	Patients	Age	Male (%)	Sampling time	Outcome
This study, 2024, Shantou and Xi’an	P	≤2.0 g/L	1,981	62	1,541 (77.8%)	The first day of admission	Long-term mortality and 30-day mortality
Sheng Yang, 2020, Beijing	R	≤4.0 g/L	243	53.0	167 (68.7%)	Within 6 h of admission	24 h death
Jun Liu, 2018, Wenzhou	R	<2.17 g/L	143	50	103 (72.0%)	On admission	In-hospital death
Ming Li, 2021, Xi’an	R	≤2.12 g/L	206	52.3/51.84	169 (82.0%)	The first day of admission	In-hospital death
Jiahui Li, 2022, Fujian	R	Not applicable	159	52.6/50.1	114 (71.7%)	Preoperative	In-hospital death
Daisuke Arima, 2021, Japan	R	<150 mg/dl	238	65.7	108 (45.4%)	Preoperative	30-day mortality

P, prospective cohort study; R, retrospective study.

## 4 Discussion

This two-center cohort study recruited 1,981 patients with AAD, including Stanford type A and B, over a period of 6-years, and shows that patients with low PFL on admission have a higher risk of mortality, especially patients with Stanford type A AAD.

The overall mortality rate was 24.6% in our study, comparable to the in-hospital mortality rate of 27.4% in the IRAD study (16). Stanford type A AAD is associated with an early mortality of 0.5% per hour while waiting for the surgery (17). In our study, overall mortality Type A AAD was 36.1% with a mean follow-up of 21.6 months, comparable to the in-hospital mortality rate of 39.1% in the previous study (18). However, Type B with uncomplicated cases are treated conservatively, showing in-hospital mortality of below to 10%. Previous study shown that 214 diagnosis chronic Type B AAD of 5-year cumulative survival rates was 79.0% (19). It was lower with overall mortality Type B AAD was 18.6% in our study. In our study may include the patients in emergency state, such as malperfusion, rupture, and refractory pain occurs, leading to decrease survival rates.

The primary discovery of our study, with a relatively substantial sample size, is that low PFL (≤2.0 g/L) on admission is independently associated with an elevated risk of mortality in patients with AAD. Liu et al. found that low fibrinogen levels (<2.17 g/L) on admission are associated with an increased risk of in-hospital mortality in Stanford type A AAD patients (7). This association remains independent of factors such as plasma D-dimer concentration, surgery, stent-grafting and type of AAD (2, 20, 21). Recently, Yang et al. also found that 143 patients with type A AAD have lower fibrinogen levels (≤4.0 g/L) and are more likely to die within the first 24 h if they do not receive surgery (6).

To validate the results of our study, we performed a meta-analysis. The meta-analysis of the included studies and our study indicated that all-cause mortality was 3.62 times higher in Stanford type A AAD patients with low PFL compared to Stanford type A AAD patients with high PFL. Previous studies solely focus on patients with type A AAD and lack of long-term follow-up. Our patient cohort and meta-analysis encompassed both Stanford type A and B AAD patients, revealing that lower fibrinogen levels are associated with higher short- and long-term mortality only in Stanford type A patients. Simultaneously, similar associations were observed in Stanford type B in for short-term mortality. Though this association were not significant difference in Stanford type B AAD patients, we could observe negative correlation trend. Therefore, low fibrinogen levels on admission may serve as a novel clinical marker for identifying high risk individuals with AAD, especially in the short-term mortality.

Fibrinogen plays a pivotal role in facilitating effective coagulation, optimizing platelet function and promoting clot formation (22, 23). The extrinsic pathway of the coagulation cascade is activated after aortic vessel wall injury and tissue factor release (24), in which fibrinogen is cleaved by thrombin to cross-linked fibrin, resulting in excessive fibrinogen consumption and the formation of thrombi, leading to a subsequent procoagulant state (11, 25–27). Previous studies have demonstrated that the amount of coagulation and fibrinolytic activation is proportionally related to the extent of the dissection (28, 29). The fibrinolytic system is activated subsequent to the coagulation cascade in patients with AAD, as evidenced by increased levels of plasma D-dimer and other fibrin degradation products (3, 29). Hemostatic abnormalities in AAD are linked to transfusion requirements, complication risk, and mortality (30). In our study higher PT-INR levels are linked to a greater risk of mortality. Similarly, Fujimori et al. have reported, recently, that an elevated PT-INR in patients with acute aortic dissection is associated with increased transfusion volume and operation time, as well as decreased 6-year survival rates (31). As a key component of blood clots, fibrinogen balance plays an important role in hemostasis and thrombosis. Relatively insufficient fibrinogen, secondary to an overactivated fibrinolytic system, may not provide sufficient coagulation factors, ultimately increasing long-term mortality. Several guidelines recommend early infusion of fibrinogen concentrate to correct coagulation disorders (32, 33). In our study, each 1 g/L increase in PFL is associated with a 18.9% decrease in 30-day mortality rate and a 11.5% decrease in long-term mortality rate. Does actively supplementing fibrinogen reduce mortality rates? One hundred five acute Stanford type A aortic dissection patients, who received intravenous human fibrinogen, had a significantly shorter operation time, lower intraoperative blood loss, and a reduced intraoperative transfusion requirement of red blood cells than the 54 patients who did not receive intravenous human fibrinogen. The patients in-hospital mortality did not reach statistical significance, however, there was a fairly marked reduction (risk ratio, 0.41; 95% CI, 0.12–1.38; *P* = 0.15) and this procedure is highly safe (13). Thus, fibrinogen supplementation may provide a new theoretical basis for the clinical treatment for AAD.

### 4.1 Strength

The strength of our study is that this is a two-center study. People from different regions were included.

## 5 Limitations

The present study has some limitations. Firstly, it should be noted that this is an observation study, and no causal relationship can be established between fibrinogen levels and mortality in patients with acute aortic dissection. Further investigations to assess the effect of fibrinogen supplementation in AAD mortality would be interesting. Secondly, while we only recorded fibrinogen levels upon admission, multiple measurements taken throughout the course of hospitalization may provide more valuable insights into the association between fibrinogen levels and prognosis. Thirdly, some other variables that are associated with the prognosis of AAD were not available for analysis, including comorbidity of organ malperfusion and hypotension (28–31). Lastly but not least, we acknowledge that the determination of the cutoff value using X-tile software is data-driven. The lack of well-defined cut-off points is a limitation in this observational study but provides a key direction for subsequent research.

## 6 Conclusion

Low PFL is a risk factor for short and long-term all-cause mortality in patients with type A aortic dissection (AAD) and short-term all-cause mortality in patients with type B aortic dissection (AAD).

## Data Availability

The original contributions presented in the study are included in the article/Supplementary Material, further inquiries can be directed to the corresponding authors.
