# Supplementary material for: Plasma fibrinogen level and acute aortic dissection prognosis—insights from a two-center cohort study
Source: Front Cardiovasc Med. 2025 Sep 23;12:1508749. doi: 10.3389/fcvm.2025.1508749 (PMC12500716; doi:10.3389/fcvm.2025.1508749)
Supplement: Supplementary file 1 [file Image1.pdf]

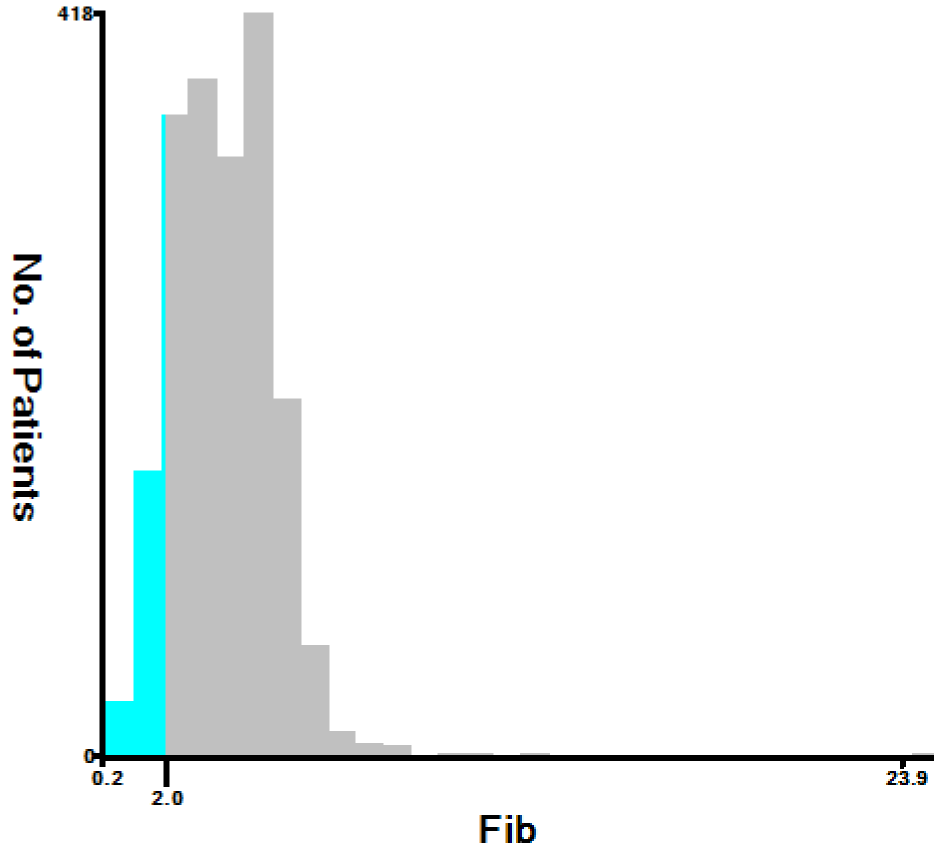

Figure S1: Determination of the cut-off score, for the plasma fibrinogen level, using the X-tile program. A cut-off score ≤ 2.0 indicates high-risk, and > 2.0 indicates low-risk.
