# Supplementary figures and images for: Plasma fibrinogen level and acute aortic dissection prognosis—insights from a two-center cohort study
Source: Front Cardiovasc Med. 2025 Sep 23;12:1508749. doi: 10.3389/fcvm.2025.1508749 (PMC12500716; doi:10.3389/fcvm.2025.1508749)

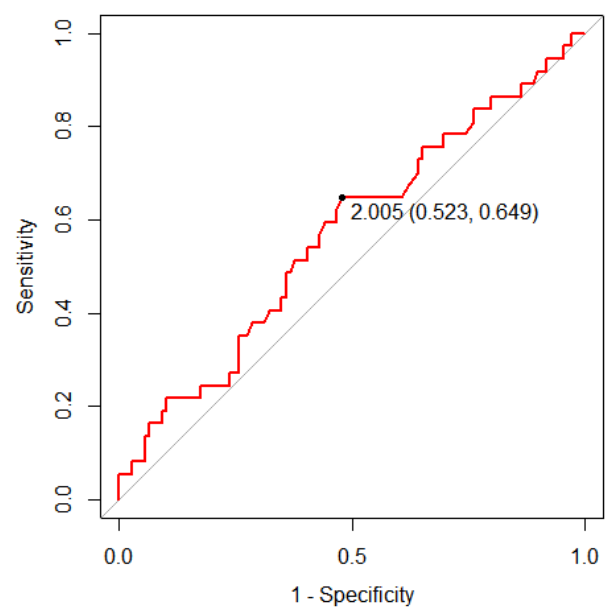

**Figure S2.** the fibrinogen cutoff value of ROC curve

Supplement: Supplementary file 2 [file Image2.pdf]

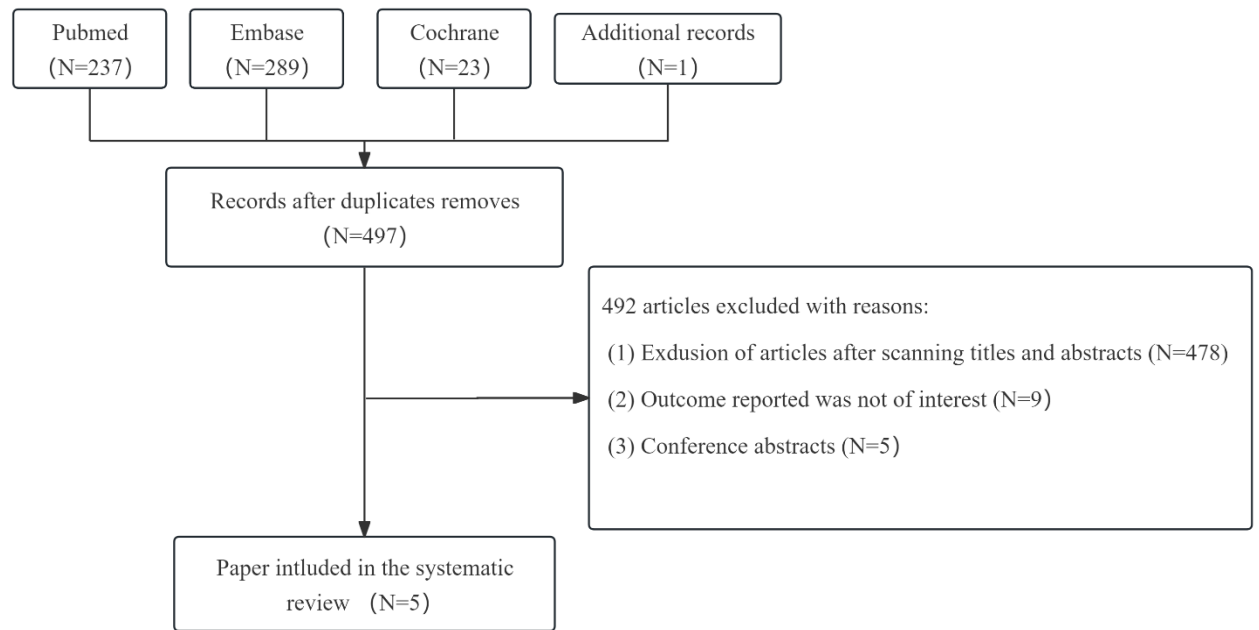

**Figure S3.** Flowchart of study selection for the meta-analysis

Supplement: Supplementary file 3 [file Image3.pdf]
