# Supplementary material for: Plasma fibrinogen level and acute aortic dissection prognosis—insights from a two-center cohort study
Source: Front Cardiovasc Med. 2025 Sep 23;12:1508749. doi: 10.3389/fcvm.2025.1508749 (PMC12500716; doi:10.3389/fcvm.2025.1508749)
Supplement: Supplementary file 4 [file Image4.pdf]

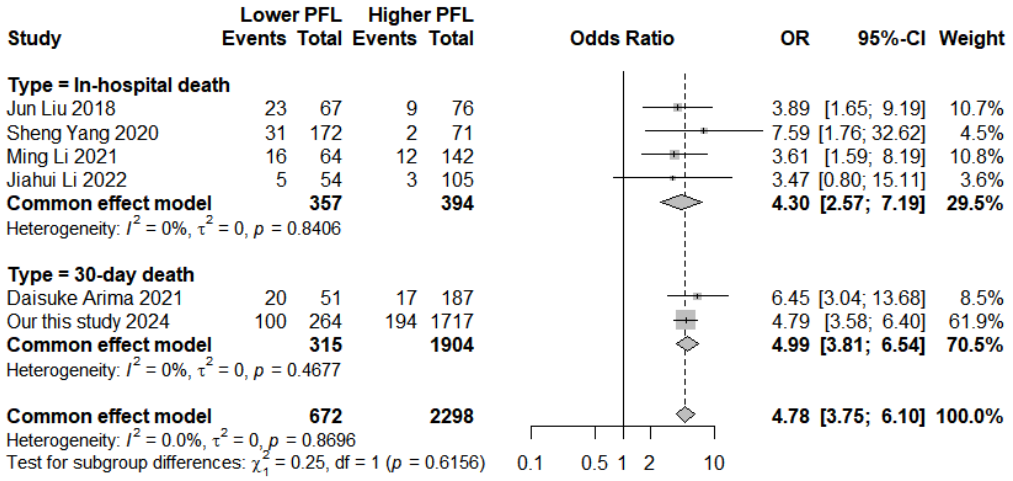

Figure S4. Forest plot of subgroup analysis for in-hospital and 30-day mortality of PFL in patients with aortic dissection.
