# Supplementary material for: Plasma fibrinogen level and acute aortic dissection prognosis—insights from a two-center cohort study
Source: Front Cardiovasc Med. 2025 Sep 23;12:1508749. doi: 10.3389/fcvm.2025.1508749 (PMC12500716; doi:10.3389/fcvm.2025.1508749)
Supplement: Supplementary file 5 [file Table1.pdf]

Table S1. Baseline characteristics of the subjects of Shantou and Xi’an hospitals.

	Overall N=1981	Shantou N=1435	Xi'an N=546	P-value
Age(years)	62(53-70)	62(52-69)	63(55-72)	<0.001
Gender Female, n(%)	440(22.2)	355(24.7)	85(15.6)	<0.001
Hypertension, n (%)	1587(80.1)	1107(77.1)	480(87.9)	<0.001
Diabetes, n (%)	549(27.7)	118(8.2)	431(78.9)	<0.001
CHD, n (%)	263(13.3)	57(4.0)	206(37.7)	<0.001
Surgery, n (%)	393(19.8)	393(27.4)	0(0.0)	<0.001
Cover stents, n (%)	729(36.8)	281(19.6)	448(82.1)	<0.001
Fibrinogen (g/L)	3.56(2.48-4.63)	2.98(2.24-3.92)	4.62(4.28-5.01)	<0.001
PT-INR	0.99(0.93-1.06)	1.00(0.94-1.07)	0.97(0.92-1.04)	<0.001
DD (μg/L)	2350(1306-4640)	3730(1840-6450)	1360.00(845-1821)	<0.001
