# Supplementary material for: Plasma fibrinogen level and acute aortic dissection prognosis—insights from a two-center cohort study
Source: Front Cardiovasc Med. 2025 Sep 23;12:1508749. doi: 10.3389/fcvm.2025.1508749 (PMC12500716; doi:10.3389/fcvm.2025.1508749)
Supplement: Supplementary file 6 [file Table2.pdf]

Table S2. Baseline characteristics between the enrolled population and the lost to follow-up population in Shantou.

	Overall<br>N=1698	Enrolled<br>N=1435	Lost follow-up<br>N=263	P-value
Age(years)	61(52-69)	62(52-69)	59(50-68)	0.065
Gender Female, n (%)	400(23.6)	355(24.7)	45(17.1)	0.009
Hypertension, n (%)	1320(77.7)	1107(77.1)	213(81.0)	0.194
Diabetes, n (%)	143(8.4)	118(8.2)	25(9.5)	0.570
CHD, n (%)	74(4.4)	57(4.0)	17(6.5)	0.098
Surgery, n (%)	446(26.3)	393(27.4)	53(20.2)	0.018
Cover stents, n (%)	346(20.4)	281(19.6)	65(24.7)	0.069
Fibrinogen (g/L)	3.00 (2.25-3.96)	2.98 (2.24-3.92)	3.05 (2.30-4.17)	0.294
PT-INR	1.00(0.94-1.08)	1.00(0.94-1.07)	1.02(0.95-1.08)	0.081
DD (μg/L)	3720(1842-6430)	3730(1840-6450)	3540(1860-6030)	0.769
